# Multidisciplinary Tracheostomy Quality Improvement in the COVID-19 Pandemic: Building a Global Learning Community

**DOI:** 10.1177/0003489420941542

**Published:** 2020-07-17

**Authors:** Chloe Swords, Lina Bergman, Rachel Wilson-Jeffers, Diane Randall, Linda L. Morris, Michael J. Brenner, Asit Arora

**Affiliations:** 1 Department of Otolaryngology – Head & Neck Surgery, West Suffolk Hospital, Bury St Edmunds, UK; 2 University of Uppsala, Uppsala, SE, Sweden; 3 St. George’s, University of London, London, UK; 4 Joe DiMaggio Children’s Hospital, Memorial Healthcare System, Hollywood, FL, USA; 5 Shirley Ryan AbilityLab, Northwestern University Feinberg School of Medicine, Chicago, IL, USA; 6 Department of Otolaryngology – Head & Neck Surgery, University of Michigan Medical School, Ann Arbor, MI, USA; 7 Department of Otolaryngology – Head & Neck Surgery, Guy’s and Saint Thomas’ NHS Foundation Trust, London, UK

**Keywords:** e-Learning, medical education, tracheostomy, patient safety/quality improvement, distance learning, webinar

## Abstract

**Objectives:**

To report experience with a global multidisciplinary tracheostomy e-learning initiative.

**Methods:**

An international multidisciplinary panel of experts convened to build a virtual learning community for tracheostomy care, comprising a web-based platform, five distance learning (interactive webinar) sessions, and professional discourse over 12 months. Structured pre- and post-webinar surveys were disseminated to global participants including otolaryngologists, intensivists, nurses, allied health professionals, and patients/caregivers. Data were collected on audio-visual fidelity, demographics, and pre- and post-tutorial assessments regarding experience and skill acquisition. Participants reported confidence levels for NICU, pediatric, adult, and family care, as well as technical skills, communication, learning, assessment, and subdomains.

**Results:**

Participants from 197 institutions in 22 countries engaged in the virtual education platform, including otolaryngologists, speech pathologists, respiratory therapists, specialist nurses, patients, and caregivers. Significant improvements were reported in communication (*P* < .0001), clinical assessments (*P* < .0001), and clinical governance (*P* < .0001), with positive impact on pediatric decannulation (*P* = .0008), adult decannulation (*P* = .04), and quality improvement (*P* < .0001). Respondents reported enhanced readiness to integrate knowledge into practice. Barriers included time zones, internet bandwidth, and perceived difficulty of direct clinical translation of highly technical skills. Participants rated the implementation highly in terms of length, ability for discussion, satisfaction, applicability to professional practice, and expertise of discussants (median scores: 4, 4, 4, 4 and 5 out of 5).

**Conclusions:**

Virtual learning has dominated the education landscape during COVID-19 pandemic, but few data are available on its effectiveness. This study demonstrated feasibility of virtual learning for disseminating best practices in tracheostomy, engaging a diverse, multidisciplinary audience. Learning of complex technical skills proved a hurdle, however, suggesting need for hands-on experience for technical mastery. While interactive videoconferencing via webinar affords an engaging and scalable strategy for sharing knowledge, further investigation is needed on clinical outcomes to define effective strategies for experiential online learning and virtual in-service simulations.

## Introduction

Patients with tracheostomies have complex needs, and when these patients suffer airway-related adverse events, they can deteriorate rapidly, risking life-threatening complications. Slow dissemination of best practices has been a major barrier to high quality tracheostomy care, despite the notable successes of multidisciplinary tracheostomy care.^1-4^ A critical knowledge gap in the United States and abroad^5^ is how to drive large-scale improvement in care. These data are critically important for quality improvement and clinical education.^6-10^ The Global Tracheostomy Collaborative (GTC), whose mission is to improve lives of individuals with tracheostomy,^4^ has spearheaded data collection efforts and dissemination of best practice.^11,12^ Yet, little is known about effectiveness of virtual approaches for engaging professionals of different disciplines. In the era of COVID-19, the stakes are greatly increased, as healthcare workers have risk of infectious transmission from aerosol generating procedures and routine care related to tracheostomy.

The COVID-19 pandemic has created massive upheaval in societal, economic, and medical systems, and in doing so it has catalyzed change in medical education and training on a scale that would have been unimaginable only several weeks ago. These changes are far-reaching and have immediate relevance to both the safety and education of frontline healthcare professionals, who range from otolaryngologist—head & neck surgeons to critical care specialists, nurses, and allied health professionals. While virtual learning in early forms dates back decades, the pandemic has led to a veritable renaissance of virtual learning—the implications of which remain largely unstudied. In the quality improvement realm, technology-enabled learning—often asynchronous in time or space—has held particular appeal, due to its promise of scalable learning that spans continents and cultures. The quality improvement efforts around tracheostomy therefore provide a compelling system for understanding the capabilities and limitations inherent in virtual learning.

In the era prior to COVID-19, we developed a virtual learning community for tracheostomy care. This article describes our experiences building a webinar series to promote interactive learning and to reach diverse audiences, with attention to the vital role of patients and family in quality improvement efforts.^13,14^ We report on a patient-centered global multidisciplinary approach to tracheostomy virtual learning. We hope that it will serve as a proof of concept to inform similar education projects in related fields, particularly with the proliferation of such methods since the onset of the global pandemic.

## Methods

### Study Design and Intervention

This prospective cohort study was conducted anonymously and in strict accordance with institutional procedures. The protocol was registered with the Institutional Review Board in accordance with the Code of Federal Regulation in compliance with University of Michigan institutional policy for conducting quality improvement projects (Study eResearch ID: HUM00171636; 45 CFR 46, 21 CFR 56). Participants were recruited via the GTC mailing list and social media venues for patient and family recruitment, inviting individuals to attend the virtual tutorials free-of-charge. There was no limit on numbers of attendees or on the study size. The GTC hosted five one-hour virtual tutorials. Table 1 details the background of the presenters and the learning objectives. The tutorials were broadcast using GoToWebinar software. After webinars, participants were able to pose questions to discussants or to the larger forum via message boards or email, as a strategy to foster the learning community.

**Table 1.** Details of Virtual Session, Including Presenter Locations, Content, and Times.

Topic	Presenters	Content and Skills
**Neonatal intensive care unit (NICU)** *1* *pm CT / 6* *pm GMT / 5* *am AEDT*	• Chief of Neonatology (MD), Massachusetts, USA • Head/Neck Surgeons, Boston and Florida, USA • Nursing Director NICU, Florida, USA	• Multidisciplinary education • Ex-utero intrapartum treatment • Neonates with congenital and acquired upper airway obstruction • Lower airway conditions
**Pediatric decannulation** *4* *pm CT / 9* *pm GMT / 8* *am AEDT*	• Head/Neck Surgeon, Michigan, USA • Nursing Clinical Care Coordinator, Michigan, USA	• Clinical readiness for decannulation • Review capping protocol • Describe the decannulation process • Prepare child and family • Post decannulation follow up
**Adult decannulation** *4* *pm CT / 9* *pm GMT / 8* *am AEDT*	• Head/Neck Surgeons, London, UK; Baltimore, USA; Washington, USA • Registered Nurse, Johns Hopkins, USA; Manchester, UK • Speech Pathologist, Australia	• Indications and contraindications • Practices as in- and out-patient • Failure and success factors • Role of multidisciplinary approach • The patients’ role • Post-decannulation education
**Patient & family perspective** *5* *pm CT / 10* *pm BST / 8* *am AEDT*	• Patient and family members • Chair of patient and family committee, GTC • Head and Neck Surgeons, Wisconsin, USA; Boston, USA	• Incorporating patients and families • Results of patient-family survey • Strategies to increase patient engagement • Opportunities with the GTC
**Quality improvement initiatives** *5* *pm CT / 10* *pm BST / 8* *am AEDT*	• Intensive Care Consultant, Manchester, UK • Head / Neck Surgeon, Manchester, UK • Nurse, Manchester, UK • Patients and family, UK	• QI at multiple levels: local department, hospital, national, international • Patient perspective

Abbreviations: AEDT, Australian Eastern Daylight Time; CT, Central Time; GMT, Greenwich Mean Time; BST, British Summer time.

### Analysis

Pre- and post-tutorial surveys were designed. Prior to dissemination, multiple healthcare professionals evaluated the survey to reduce bias in language and content. In order to optimize stakeholder representation, physician, allied health professional, and patient and family input was obtained. Participants completed the anonymized surveys voluntarily via email. Lack of evaluation did not affect eligibility to participate in future sessions. There were no financial incentives.

Survey participants scored audio and visual fideltiy using a 1 to 5 Likert scale. Anchors at 1 and 5 corresponded to “strongly disagree” and “strongly agree,” respectively. An overall average baseline quality score of 4, on a 5-point Likert scale, was set *a priori* as the minimum score for inclusion.

Participants reported confidence level before and after each virtual session. Impact was evaluated by assessing confidence of participants regarding their tracheostomy-related knowledge and ability to apply that knowledge for the content covered in each tutorial. Assessments were conducted before and after the tutorials using a 5-point Likert scale. Anchors at 1 and 5 corresponded to “not very confident” and “very confident,” respectively. Multiple questions on each topic were posed to participants covering a variety of different themes. For the purpose of this article, a theme was identified as an overarching area of particular importance or concern, such as communication or practical skills. Topics were defined as the subject matter of the webinar, such as neonatal tracheostomy. This “overlapping wedge” concept, utilizing horizontal (topics) and vertical (themes) integration, is often used in medical education to improve learning experiences for students.

Median values were calculated. Immediate participant reaction and satisfaction with the program were also evaluated using a 5-point Likert scale. Participants were asked to provide free-response answers regarding specific plans for their future practice as a surrogate for long-term impact of the program.

### Data Analysis

Descriptive statistics were summarized for course evaluation. Overall confidence score was calculated. Values were interpreted as being independent, as pre- and post-survey responses were anonymized. A Mann-Whitney U-test was used to compare differences in confidence values between pre- and post-tutorial scores. Values were calculated for each tutorial and for key themes (technical skills, communication, lifelong learning, clinical assessments, clinical governance). Statistical significance was set at *P*-values less than 0.05. To account for missing data, a non-matched statistical analysis method was employed. The statistical package GraphPad Prism 7.0 was used for data analysis. Interquartile ranges (IQR), reported for assessments of educational impact, correspond to the difference between the largest and smallest values in the middle 50% of each data set.

## Results

### Demographics

Table 2 illustrates participation in virtual tutorials, detailing data on individuals who pre-registered, attended the live tutorial, or watched the recording at a later date (asynchronous viewing). A total of 328 surveys were completed: 225 before and 103 after the tutorials. Survey response rates of those attending the live tutorial were 51% before and 23% afterwards. Table 3 illustrates the demographic characteristics of survey respondents. Participants from 197 different institutions responded to the surveys; the majority were from North America. A total of 22 countries were represented. Individuals from multi-disciplinary team frequently attended.

**Table 2.** Registrants and Attendees. The live attendees number is determined automatically by the webinar platform software and does not account for those who viewed the webinar as a group (extracted in January 2018).

Topic	Date	Number of Registrants	Number of Attendees (live)	Number Viewed the Recording at Later Date
**Neonatal intensive care unit**	November 2016	135	67	111
**Pediatric decannulation**	January 2017	157	89	173
**Adult decannulation**	March 2017	304	156	285
**Patient and family perspective**	May 2017	199	86	94
**Quality improvement initiatives**	July 2017	121	43	109

**Table 3.** Demographic Characteristics of Survey Responders.

	Before Webinar	After Webinar
**Participants, n**	225	103
NICU	34	23
Pediatric	40	19
Adult	86	34
Patient and family	49	20
Quality improvement	16	7
**Age, mean (range)**	42.4 (24-77)	43.4 (27-71)
**Female: Male**	167:31	84:18
**Number of institutions**	136	61
**Profession, n (%)**		
Nurse	35 (15.6)	17 (16.5)
Speech & language pathologist	55 (24.4)	20 (19.4)
Physiotherapist & Respiratory therapist	33 (14.7)	17 (16.5)
Doctor	40 (17.8)	27 (26.2)
Social worker	5 (2.2)	2 (1.9)
Non-clinical	26 (11.6)	10 (9.7)
Other	31 (13.8)	10 (9.7)
**Country, n (%)**		
USA, Canada	115 (51)	61 (59.2)
Australia, New Zealand	47 (20.9)	22 (21.4)
UK	42 (18.7)	14 (13.6)
Other European	10 (4.4)	3 (2.9)
South America	1 (0.4)	1 (0.9)
Asia	10 (4.4)	2 (1.9)

### Audio-Visual Quality

**Figure 1.**
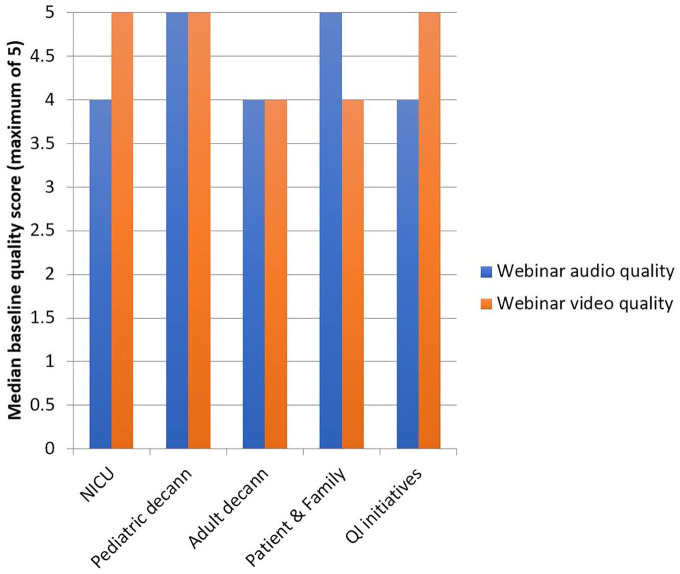
Audio and video quality. An overall median score of 4 out of 5 was set *a priori* as the minimum score for inclusion. Abbreviations: decann, decannulation; NICU, neonatal intensive care unit; QI, quality improvement.

All tutorials met the minimum baseline score for inclusion in analysis (Figure 1).

### Overall Response to Virtual Learning

Participants rated the course highly in terms of length, opportunity for discussion, overall satisfaction, applicability to professional practice, and expertise of discussants (median scores: 4, 4, 4, 4 and 5). Participants provided suggestions regarding areas that they enjoyed or felt could be improved (detailed below). In terms of content, participants requested additional time for discussion and utilization of case scenarios. Queries were collated and addressed by discussants and other experts in tracheostomy care, making regular use of GTC forums to facilitate additional discourse on practical aspects of tracheostomy-related care.

### Educational Impact

#### Impact by tutorial

**Figure 2.**
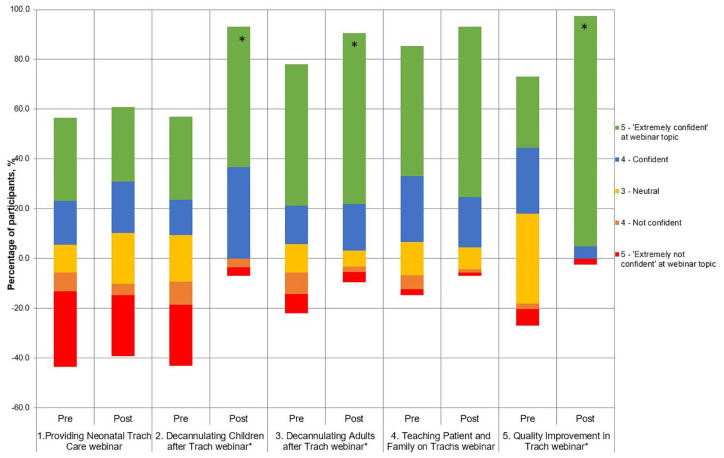
Participant confidence regarding mastery of education content covered, measured before and after the five virtual tutorials: Diverging stacked bar chart. *Those tutorials with statistically significant improvement in confidence before and after (*P* < .05).

A shift in participant’s responses towards being “confident” and “extremely confident” was observed in all five tutorials (Figure 2). The median values of Likert responses are presented; multiple domains were assessed to reflect different aspects. Significant improvements were demonstrated following the pediatric decannulation tutorial (pre-course median 3.25 [IQR3.125] vs post-course median 4.5 [IQR1]; *P* = .0008), adult decannulation tutorial (median 4.5 [IQR2] vs 5 [IQR0.5]; *P* = .04), and quality improvement tutorial (median 3.5 [IQR1] vs 5 [IQR0]; *P* < .0001).

#### Impact by themes

**Figure 3.**
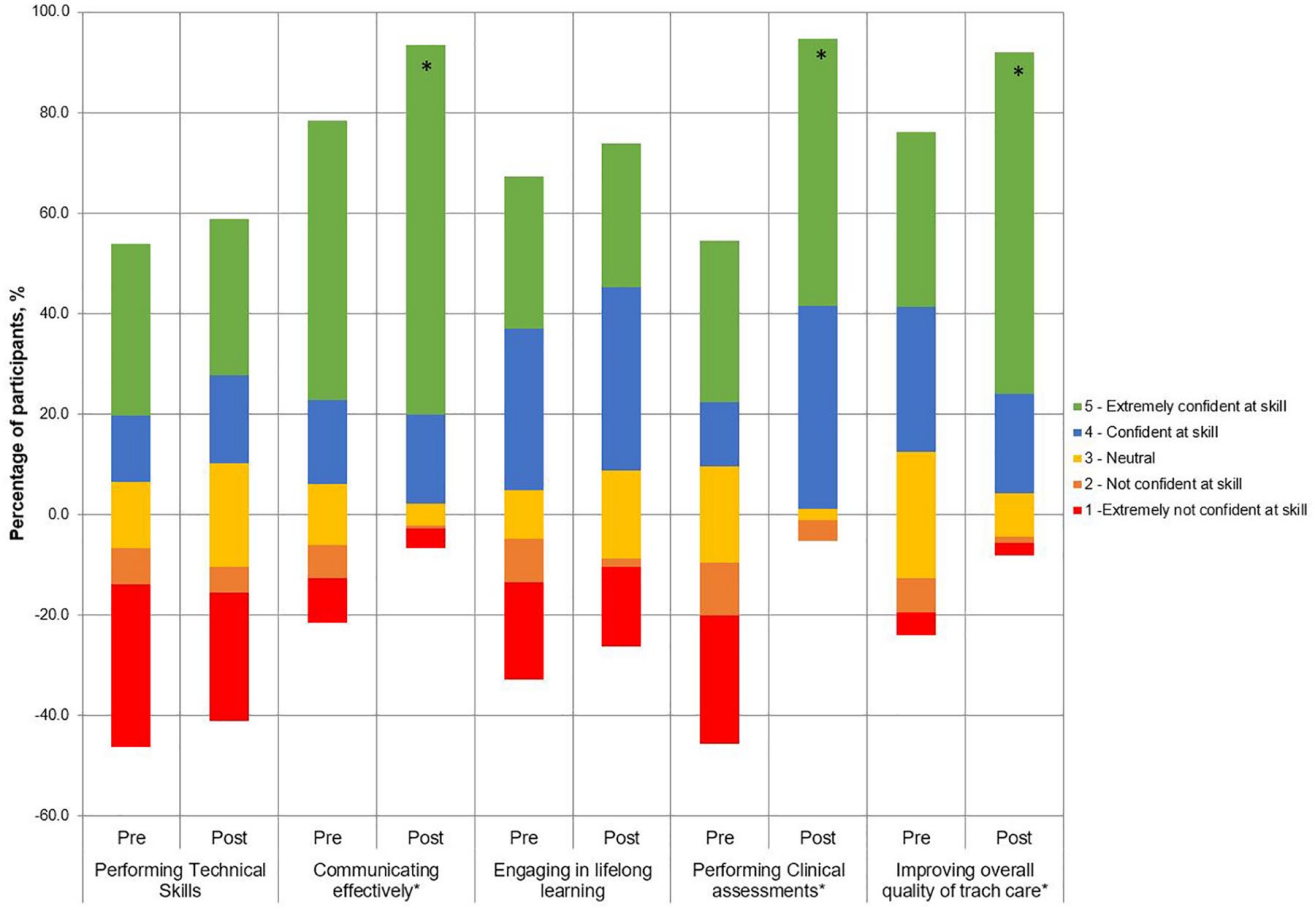
Participant confidences in core competency areas of education were measured before and after the five virtual tutorials: Diverging stacked bar chart. *Those tutorials with statistically significant improvement in confidence before and after (*P* < .05).

A shift in participant’s responses towards being “confident” and “extremely confident” was seen in the five themes (Figure 3). Significant improvements were observed for communication (pre-course median 4 [IQR2] vs post-course median 5 [IQR1]; *P* < .0001), clinical assessments (median 3 [IQR4] vs 5 [IQR1]; *P* < .0001), and clinical governance (median 4 [IQR2] vs 5 [IQR1]; *P* < .0001).

### Change in Behavior of Daily Clinical Practice

Several participants identified specific changes in behavior relating to daily practice. Respondents conveyed thinking more in terms of the multidisciplinary team and of sharing information with others. Most respondents reported enthusiasm to engage in quality improvement initiatives at their hospital. While some participants felt that they would be more confident in carrying out practical skills, some respondents indicated residual reservations about assisting in technical procedures and ability to carry out the algorithm recommended for achieving decannulation.

### Evolution of the Series

A number of metrics were analyzed to facilitate ongoing progress of the sessions and learning community. Early feedback from participants noted concerns of poor audio-visual content, which cut out several times. Following that first session, a debriefing meeting was conducted. The committee concluded that, in addition to rehearsing presentation content, it was equally important to rehearse the technology. This measure enabled all presenters to ensure compatibility of audio-visual systems with the software. Audio-visual interruptions were also minimized by hardwiring the internet and using phones for audio instead of computer microphones.

Further ongoing assessments revealed that participants who registered on the day of the session were more likely to attend than those who registered in advance. Partly this trend was associated with likelihood of viewing the session at a later time. Nonetheless, this insight enabled the planning team to increase the live attendance rates by focusing advertisements in days before presentations and providing targeted reminder emails to those registered in advance (1 week, 5 days, 1 day, 1 hour before), leading to a pronounced spike in attendees at the following session.

Timing was another significant factor. The first session was held at 2 pm EST, corresponding to presenters’ availability. However, participants from Australia signaled that this corresponded to a 5 am meeting in Sydney. Following discussions with the education committee, 5 pm EST, 9 pm Greenwich Mean Time and 8 am Australian Eastern Daylight Time was agreed. As Western Australia, Queensland and Northern Territory do not observe daylight saving, this corresponded to 10 pm British Summer Time from March to October. It was decided that 10 pm in the United Kingdom was preferable to 7 am in Australia. Reports from participants in Australia indicated that attending sessions as a group breakfast meeting was well received.

## Discussion

Our data, spanning 22 countries and 197 institutions, reflects experiences across a spectrum of professionals involved in providing multidisciplinary tracheostomy care. The significant improvements around perceived knowledge and skills support virtual education in otolaryngology, with the caveat that a lack of impact was observed in technical skills. This finding highlights the importance of hands-on and experiential learning. The finding of pre- and post- webinar improvement is in keeping with prior studies evaluating virtual learning, where healthcare professionals had gain in knowledge comparable to learners receiving traditional teaching (Stain *p* = 0.65; Joshi *p* = 0.6; Bertsch *p* = 0.66).^15-17^ Black *et al* performed post-tutorial survey polls, which demonstrated that most survey respondents (71%, n = 89) believed that virtual learning was effective for information sharing and knowledge acquisition.^18^

Preparing surgeons and other healthcare professionals adequately enhances patient safety and operational efficiencies.^4^ The global engagement in these efforts to improve tracheostomy care is heartening. Since completion of the current data series, the interest in these virtual forums has increased an order of magnitude, with over 1500 registrants in a recent session. Otolaryngologists, as leaders in airway surgery, can have more impact if they embrace technology. The current COVID-19 pandemic is placing a worldwide strain on healthcare, and it becomes increasingly important to encourage global collaborative learning and sharing of information. The number of tracheostomies performed in critically ill patients will likely increase during the pandemic. There are new considerations such as airway safety and timing of the procedure,^19,20^ as well as ensuring healthcare worker safety.^21^ Virtual learning is a valuable medium for rapidly sharing clinician expertise around global strategies to reduce aerosolisation as well as timely topics regarding healthcare disparities.^22^ During this period, the GTC has run six webinars involving thousands of attendees with broad engagement on timely topics ranging from healthcare worker safety and public health to healthcare disparities. The work described above acted as the stepping-stone to enable this accomplishment.

### Advantages of Virtual Learning

Participants in this tutorial series demonstrated high satisfaction with the program, and this finding is significant for a couple of reasons. First, individuals who have a positive experience are more likely to return for future sessions, as demonstrated by the growing engagement in these sessions in recent weeks. A more nuanced reason relates to our goal of cultivating a learning community. To do so, participants must develop a sense of camaraderie and trust. Anecdotally, we can report instances where bonds have formed or strengthened spanning continents, between fellow otolaryngologists and other specialists who see common problems and find common solutions.

Medical education, whether in-person or virtual format, ideally involves bidirectional sharing of ideas. Live virtual learning is highly conducive to this goal. The “question and answer” period at the end of each session allowed participants to share their own ideas and pose questions to the speakers, enabling interactive participant engagement. A collaborative, team-based approach to e-learning facilitates sustainable, responsive and multidisciplinary developments within a field that is constantly changing and evolving.^23^

Medicine is collaborative,^24^ and increasingly focused on experiential learning.^25^ Virtual learning has tremendous potential for collaborative multidisciplinary efforts. The needs of patients with tracheostomies frequently traverse traditional cross-specialty boundaries, requiring collaboration and communication between multiple teams. The series was attended and taught by various specialties, as well as caregivers and community teams. The background of speakers for each session included representation from a minimum of two countries, so as to facilitate an international perspective. Virtual tutorials, such as those conducted in this study, afford participants an opportunity to interact with worldwide experts in a variety of professions as well as patients. Interprofessional education has been shown to improve students’ knowledge of tracheostomy care and professional roles.^26,27^ Patient involvement in interprofessional education may be beneficial in fostering person-centeredness, particularly in undergraduate students.^28,29^ The importance of interprofessional education in health professions training is increasingly recognized through new accreditation guidelines,^30^ and is being implemented in medical curricula as an effective learning method.^31^ A recent systematic review highlighted positive results in students’ attitudes towards learning and interprofessional collaboration; but highlighted concerns related to difficulties of implementation.^31^ More insight is required into the design of effective interprofessional education curricula, particularly in the context of virtual training.

### Limitations Inherent in Virtual Learning

When technical skills are critical, as in tracheostomy care, virtual learning is ideally combined with in-person simulation, in-services, or other practical training.^32^ A randomized controlled trial (n = 168) evaluated different versions of a training program for physician-patient or teacher-parent communication.^33^ The results indicated that a combined method of both e-learning and role play showed the greatest effect; interestingly e-learning proved more effective than role play alone in this particular study.^33^ A large number of individuals viewed the tutorials in recorded format, rather than the intended live version. This highlights versatility but also difficulties inherent in learning across continents. Survey responses were only requested from individuals who participated in the live activity.

A final limitation relates to fidelity. Satisfactory hardware is crucial for audio and video quality.^34,35^ There were rare instances of frustrations attributed to loss of audio-visual display, similar to other studies.^16,36^ In patients with tracheostomies using speaking valves, audibility was also sometimes degraded. Further difficulties may arise from suboptimal device settings. It is advisable to check equipment beforehand and to use mute functions to minimize distractions. Older devices are prone to difficulty. Such issues may be more likely to occur in resource-limited settings. Other initiatives have addressed methods to improve tracheostomy care in such settings, including publishing an electronic open-access online database of tracheostomy care.^37^

When possible, a fixed/wired Internet connection is preferable to Wi-Fi and mobile networks, which have fluctuating bandwidth. Headsets with USB plugs are beneficial, as high quality inbuilt microphones are uncommon. Echo-suppressing microphones are required when using speakers. Less technologically savvy users may not realize that computers usually default to lower fidelity built-in microphones.

### Evaluation of the Series

Future work is needed to assess how virtual tutorials can be combined with direct skills training to drive improvements in outcomes, perhaps by running a tandem standardized practical skills tracheostomy training program for participants following tutorials and addressing relevant skills. Table 4 illustrates candidate outcomes for monitoring. Accredited individuals could run sessions in each country. Additionally, more readily achieved future directions involve refining strategies that allow custom tailoring content to a culturally, geographically, and professionally diverse audience.

**Table 4.** Outcomes Monitoring Requirements.

Suggestion	Comment
**Demographics**	Information regarding study population should be used to contextualize presentation and responses.
**Educational impact**	Pre and post-webinar quizzes is desirable, ultimately with long-term evaluation of impact on professional practice.
**Audio and visual quality**	Fidelity of technology is crucial, as participants can readily disengage if there are unnecessary distractions.
**Participant satisfaction**	Positive responses reflect learning outcomes and can be measured using, for example, 5-point Likert scale.

It is important that outcomes are evaluated in a structured fashion to ensure monitoring of the learning session. A systematic review of technology-enhanced learning for healthcare professionals found that only 64% of 417 studies presented any form of validity evidence.^26^ These limitations restrict conclusions, and so from the outset we sought to build rigor into analysis for the present study by adopting the Kirkpatrick methodology. This validation process was developed in industry and is supported by the Best Evidence Medical Education initative.^27^ The four-part model comprises a series of evaluation levels, to appraise increasingly complex outcomes. However, our experience with this model for outcome monitoring for webinars was challenging, and despite demonstrating improvements in confidence and participant assigned intent to change behaviors in response to the webinars, any such impact is speculative. We therefore have only been able to conclusively demonstrate a benefit in Kirkpatrick level 1.

Further studies are required to determine which aspects of healthcare education are most conducive to virtual learning. There is a need for clear reporting of methodology to ensure reproducibility, to validate outcomes, and to demonstrate sustained impact. The improvement in confidence across many domains and topics found in this study is an encouraging result, as there is a growing body of evidence documenting the importance of self-efficacy and its relation to agency and performance.^38^ Such work on self-efficacy has been applied to surgical skill acquisition,^39^ and the significance of nursing comfort level as a barrier to tracheostomy care has also been reported.^40^

### Study Limitations Relating to Data Collection

The number of participants in each session is unknown and easily underestimated. For example, participants from Austin Health, the major health system in Melbourne, Australia, shared that many of its clinicians viewed webinars in a large theater, which is counted as a single registrant with one survey response. Furthermore, the 23% response rate for the follow-up voluntary questionnaire indicates that a preponderance of post-tutorial data was un-captured and introduces a selection bias. One encouraging finding regarding representative sampling is that the proportion of participants completing the survey was uniform across dimensions of country, profession, age, gender, and parent versus adult. These data compare favorably to other electronic survey data in the otolaryngology quality improvement literature, which typically report response rates below 20%.^41,42^

## Conclusion

The COVID-19 pandemic has changed the world permanently, including modifying the fabric of medical education and clinical care. The pervasive use of technology for virtual communication that has been a necessity in a time of social distancing will have a lasting effect across the continuum of medical education, residency, and continuing medical education. Instructional lectures aiming to improve tracheostomy and airway knowledge deficits^43^ that were previously delivered face-to-face are one such option that could be converted to virtual learning. The Association of American Medical Colleges (AAMC) has created a free and open resource repository to facilitate sharing and disseminating educational approaches. Even once the pandemic subsides, many of the innovations that were effectively shoehorned into our daily lives are here to stay, with hopefully salutary effects on otolaryngology’s educational, clinical, and quality improvement operations. While the experience presented herein with virtual learning was initiated before the pandemic, it nonetheless affords a window into innovations ranging from resident education to virtual care to virtual clinical learning.

Virtual learning allows broader reach than local forums and offers interaction not available in independent online learning. Today’s millennial medical students and practitioners are highly receptive to incorporating technology in learning.^44^ Literature on mentoring of millennials emphasizes benefits of less hierarchical interactions and flexible approaches, such as micro-mentoring, reverse-mentoring, and adaptable engagement.^45^ Many aspects of otolaryngology require embracing multidisciplinary approaches, and engagement with technology positions otolaryngologists as innovators who can lead in promoting collaborative learning. The lessons garnered from this endeavor are not only of practical value in preparing for tracheostomy during the COVID-19 outbreak, but also can enhance forward looking quality improvement and educational initiatives.
